# The 2010 Outbreak of Cholera among Workers of a Jute Mill in Kolkata, West Bengal, India

**DOI:** 10.3329/jhpn.v29i1.7561

**Published:** 2011-02

**Authors:** Prakash Mridha, Asit K. Biswas, R. Ramakrishnan, Manoj V. Murhekar

**Affiliations:** ^1^ Field Epidemiology Training Programme, National Institute of Epidemiology, R-127, Tamil Nadu Housing Board, Ayapakkam, Ambattur, Chennai 600 070, India; ^2^ Strategic Planning and Sector Reform Cell, Department of Health and Family Welfare, Government of West Bengal, West Bengal, India

**Keywords:** Case-control studies, Disease outbreaks, Cholera, Vibrio cholerae, Water pollution, Water supply, India

## Abstract

On 10 March 2010, an outbreak of diarrhoeal disease was reported among workers of a jute mill in Kolkata, West Bengal, India. The cluster was investigated to identify the agent(s) and the source of infection and make recommendations. A suspected case of cholera was defined as having >3 loose watery stools in a 24-hour period and searched for case-patients in the workers’ colony. The outbreak was described by time, place, and person, and a case-control study was conducted to identify the source of infection. Rectal swabs were collected from the hospitalized case-patients, and the local water-supply system was assessed. In total, 197 case-patients were identified among 5,910 residents of the workers’ colony (attack rate 3.33%). Fifteen of 24 stool samples were positive for *Vibrio cholerae* O1. The outbreak started on 7 March, peakedon 11 March, and ended on 16 March 2010. Compared to 120 controls, 60 cases did not differ in terms of age and socioeconomic status. Drinking-water from the reservoir within the mill premises was associated with an increased risk of illness [odds ratio: 26.7, 95% confidence interval (CI) 11.4-62.6) and accounted for most cases (population attributable risk percentage=82%, 95% CI 70.8-92.9). An outbreak of cholera occurred among workers of the jute mill due to contamination of the drinking-water reservoir. It occurred within a few days of re-opening of the mill after the workers’ strike. Health authorities need to enforce disinfection of drinking-water and regularly test its bacteriological quality, particularly before re-opening of the mill after the strike.

## INTRODUCTION

Cholera, an acute infectious disease caused by toxigenic strains of *Vibrio cholerae* serogroup O1 and O139, is transmitted through contaminated water and food (1, 2). The enterotoxin produced by *V. cholerae* stimulates the secretion of water and electrolytes in the intestinal lumen. Patients with chole-ra may suffer from acute watery diarrhoea, vomiting, and dehydration but rarely present with fever (1, 2). Cholera continues to be an important health problem in India. Sixty-eight outbreaks of cholera were reported from the country during 1997-2006, affecting more than 200,000 cases with 823 deaths. Nearly one-fourth of the outbreaks and 42% of deaths were from the Indian state of West Bengal. *V. cholerae* O1 belonging to the El Tor biotype is the most common serogroup in the country while the frequency of serogroup O139 has declined considerably over the past few years (3).

On 10 March 2010, the local Councilor of Ward no. 80 of the Kolkata Municipal Corporation (KMC), West Bengal, India, informed the corporation health authorities about a cluster of diarrhoeal disease**s** among the workers of a jute mill. Several of these case-patients were admitted to the Infectious Disease Hospital, Kolkata. We investigated this outbreak with the objectives of (a) estimating the magnitude, (b) identifying the aetiological agent and the source of infection, and (c) guiding the prevention and control measures.

## MATERIALS AND METHODS

### Descriptive epidemiology

We reviewed the diarrhoeal disease surveillance data from the office of KMC for the previous five years to confirm the existence of the outbreak. We defined a suspected case of cholera as occurrence of three or more loose stools in a 24-hour period among the residents of a jute mill colony since 5 March 2010. A suspected case with isolation of *V. cholerae* was considered a confirmed case. This case definition was consistent with the case definition of the Integrated Disease Surveillance Project (IDSP) in India (4). We searched for case-patients by door-to-door visits in the workers’ colony and collected information about the sociodemographic details of all the residents and clinical details, date of onset of illness, and place of residence from the case-patients.

We calculated the incidence by age and sex using population denominators collected during the house-to-house search. An epidemic curve was constructed to describe the distribution of case-patients over time. The cases were plotted on a spot-map to understand their geographical distribution. To formulate the hypothesis, we interviewed some case-patients to collect information about possible exposures, including drinking-water, drainage system, hygiene practices, and whether there was any common festival or mass gathering within a week of onset of the outbreak.

### Environmental investigations

We visited the jute mill and the mill colony to ins-pect and review the water supply and sanitation system. Drinking-water samples were collected from all the water sources within the mill and also from the corporation tap-water sources in the workers’ colony. Their bacteriological quality was tested.

### Laboratory investigations

Rectal swabs were collected from a sample of case-patients admitted to the Infectious Disease Hospital during the course of the outbreak. Rectal swabs were sent to the National Institute of Cholera and Enteric Diseases (NICED), Kolkata, for microbiological investigations. Water samples taken from inside the mill and workers’ colony were tested for coliforms by membrane-filtration technique at the water analysis department of the KMC.

### Analytical epidemiology

We conducted an unmatched case-control study to test the hypothesis that the outbreak was associated with drinking-water from the reservoir in the mill premises. Assuming the confidence level of 95%, power of 90%, case:control ratio of 1:2, and 40% of the controls drinking water from this source, we calculated the sample-size of 60 cases and 120 controls to detect a minimum odds ratio of 3. We included all the case-patients admitted to the hospital as cases and randomly selected two healthy persons from the employee register and staying in the workers’ colony as controls. Using a pre-tested, close-ended structured questionnaire, we collected information regarding general characteristics, clinical features, and potential risk factors.

Univariate and multivariate analyses were conducted using the Epi Info software (version 3.3.2, 2005) of the Centers for Disease Control and Prevention (CDC), Atlanta, GA, USA. We calculated odds ratios (ORs) and their 95% confidence intervals (CIs) for different exposures and estimated the attributable fraction and population attributable fractions using the OpenEpi software (version 2.2, 2007).

## RESULTS

### Descriptive epidemiology

Of the 5,910 residents in the workers’ colony, 3,057 (52%) were male. We identified 197 case-patients with an overall attack rate of 3.3%, with no death. All the 197 case-patients were male (attack rate 6.4%) and were working in the mill while none of the family members of the workers developed the illness. The attack rate was the highest (6%) in the age-group of 45-54 years (Table 1). Of the 197 case-patients, 92% had dehydration, 89% had vomiting, and 51% were hospitalized. Cases started occurring from 7 March, peaked on 11 March, and the outbreak subsided on 16 March 2010 (Fig.). The spot-map of the residential area did not show any clustering (data not shown).

**Table 1.** Distribution of suspected cholera cases by age in the jute mill workers’ colony, Kolkata, West Bengal, India, March 2010

Age-group (years)	Population	Cases	Attack rate (%)
0-14	1,271	0	0.0
15-24	589	17	2.9
25-34	959	32	3.3
35-44	1,167	57	4.9
45-54	1,113	66	5.9
55-64	591	25	4.2
≥65	220	0	0.0
Total	5,910	197	3.3

**Fig.**
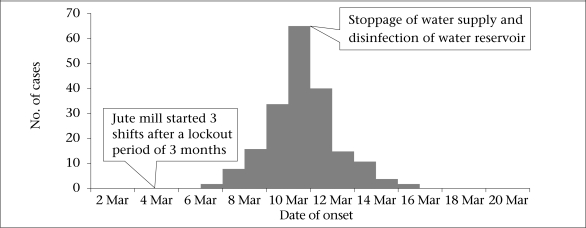
Distribution of suspected cholera cases by date of onset, Kolkata, West Bengal, India, March 2010

### Laboratory investigations

*V. cholerae* O1 El Tor Ogawa was isolated from 15 of the 24 rectal swabs. All the five water samples from the water sources inside the factory (one from theoverhead tank, one from the drinking-water reservoir, and three from water-taps, coming from the overhead tank) showed coliforms, indicating faecal contamination. On the other hand, all the four water samples from the KMC water-taps in the workers’ colony had no coliforms.

### Environmental investigations

The jute mill, situated by the side of the Hooghly river, existed for more than 100 years. The mill received water from two sources: (a) the underground water, which was stored in an overhead tank, was primarily used for industrial purposes and for domestic use, e.g. washing, bathing and (b) water supplied through the KMC was used for drinking purposes. This water was stored in the reservoir in the mill premises and distributed to the workers through three taps. Water from the KMC was supplied daily in adequate quantity. This reservoir also received water from the overhead tank that contained the underground water. The workers’ colony received water from the KMC.

The jute mill was closed during 14 December 2009–12 February 2010 due to the workers’ strike. It was re-opened on 13 February 2010 and became operational on 5 March 2010, when more than 1,500 workers joined the work. Three weeks before the start of its operations, the mill management undertook the maintenance of mechanical equipment with a few workers (working for short duration). The drinking-water reservoir, however, was not cleaned or disinfected.

### Analytical epidemiology

The cases and controls did not differ by age (mean age of cases and controls: 41.7 and 41.8 years res-pectively, p=0.99), education level (proportion of cases and controls educated up to primary level: 64% and 67% respectively, p=0.53), and income (median monthly income of cases and controls: Rs 5,500, p=0.67). Compared to the controls, more case-patients drank water from the drinking-water reservoir within the compound of the mill during the duty hours (OR=26.7, 95% CI 11.4-62.6). This exposure accounted for the majority of cases (attributable risk among exposed=96.3%, 95% CI 91.2-98.4 and population attributable risk=82%, 95% CI 70.8-92.9). Certain practices, such as washing hands before taking food and after defaecation, were associated with a lower risk of developing the illness (Table 2). On multiple logistic regression, the adjusted OR associated with drinking-water from the reservoir within the mill premises was 12.1 (95% CI 4.3-34).

**Table 2.** Distribution of cases and controls according to selected exposure variables in the study jute mill, Kolkata, West Bengal, India, March 2010

Exposure variable	Cases (n=60)		Control (n=120)		Univariate odds ratio (95% CI)	Adjusted odds ratio (95% CI)
	No.	%	No.	(%)		
Reservoir within mill compound as the main source of drinking-water during work	51	85	21	17.5	26.7 (11.41-62.6)	12.1 (4.3-34.0)
Consumption of food from local vendor	6	10	11	9.2	1.1 (0.37-3.1)	2.6 (0.5-14.7)
Regular washing of hands before food and after defaecation	11	18.3	107	89.2	0.03 (0.01-0.07)	0.06 (0.02-0.17)

CI=Confidence interval

### Control measures

After the onset of the outbreak, the factory authorities stopped the use of drinking-water from the reservoir within the mill premises. The reservoir and the overhead tank were disinfected on 11 March 2010, and the disinfection process was continued for three consecutive days.

## DISCUSSION

An outbreak of cholera occurred in the jute mill in Kolkata within days after the factory was re-opened after the workers’ strike. Our investigations pointed to the contamination of the drinking-water reservoir within the factory premises as the source of the outbreak, with more than 95% of the cases being attributed to this exposure. Prompt hospitalization of the cases, immediate discontinuation of the use of the drinking-water reservoir within the mill premises and its disinfection stopped the outbreak and prevented secondary cases in the workers’ colony.

Several factors supported our finding that the outbreak was due to contamination of the drinking-water reservoir within the mill premises. First, all the cases were identified among the factory workers, with no secondary cases among the residents of the workers’ colony. Second, the shape of the epidemic curve supported exposure to a common source. Third, the consumption of drinking-water from this source was associated with an increased risk of illness, with more than 95% of the cases being attributed to this exposure. Fourth, the water samples from the drinking-water reservoir and overhead tank containing underground water were found to be faecally contaminated. Although we did not attempt to isolate *V. cholerae* from water, the findings of our investigation support our hypothe-sis that reservoir water was contaminated with *V. cholerae*. It appeared most likely that the drinking-water reservoir was contaminated by underground water supplied through the overhead tank.

Outbreaks of cholera are common in West Bengal. For example, 16 outbreaks of cholera were reported during 1996-2007 from this state (3). These outbreaks were reported both from urban and rural areas and were due to contamination of drinking-water sources, including wells [Das PK, Biswas A, Ramakrishnan R, Hutin Y, Gupte M. Unprotected wells continue to cause cholera outbreaks in West Bengal, India, 2006. Presented at: the Fourth South-East Asia and Western Pacific Bi-regional TEPHINET Scientific Conference, 26–30 November 2007, Taipei, Taiwan, China], ponds [Rudra S, Ramakrishnan R, Hutin Y, Gupte M. A cholera outbreak in a village of West Bengal, India, 2006: the danger of using ponds for soiled clothes disposal. Presented at: the Fourth South-East Asia and Western Pacific Bi-regional TEPHINET Scientific Conference, 26–30 November 2007, Taipei, Taiwan, China], and piped water (3, 5, 6). The present outbreak is the first one reported in a mill setting in West Bengal. Outbreaks of cholera due to contamination of drinking-water sources have been reported in other occupational settings, such as gold mines and oil rigs (7, 8).

According to the Factories Act of India, it is mandatory that every factory has effective arrangements for provision and maintenance of suitable drinking-water points, with all such points legibly marked as ‘drinking-water’ in a language understood by the majority of workers employed in the factory (9). The arrangements for drinking-water were adequate in the jute mill, although there was no signage for ‘drinking-water’. Before making the factory operational after the workers’ strike, the mill management undertook maintenance of mechanical equipment. This maintenance work was done by a small number of management staff and workers, and none of them developed illness as they did not consume water from the reservoir. The factory management, however, paid no attention to cleaning or disinfecting the water reservoir in the mill, thereby leading to an outbreak.

### Limitations

Our investigation had certain limitations. First, we identified contamination of the drinking-water reservoir in the mill premises as the source of outbreak. This contamination might have occurred due to the mixing of underground water. We, however, were not able to explain how the underground reservoir was contaminated. Second, we only included admitted case-patients in the study. These case-patients are likely to recall more about water-consumption history compared to the healthy controls. However, such bias would be very small as the study subjects and the trained investigators were unaware of the hypothesis under investigation.

### Conclusions

A large outbreak of cholera occurred among workers of a jute mill in Kolkata due to contamination of the water reservoir. This contamination probably occurred through the underground water supplied from the overhead tank. We made a number of recommendations and engaged several interventions. First, we educated the mill workers not to drink the reservoir water till the outbreak was over. Second, we disinfected the reservoir and recommended its periodic chlorination. Third, the connection between the reservoir and the overhead tank was closed and recommended to supply water for drinking from the city corporation only. The findings of our investigations would also be useful in preventing similar outbreaks in the state where jute textile manufacturing is the most prominent industry. The health authorities from the KMC and the Department of Health need to ensure that factory management disinfects the drinking-water reservoirs and tests these for their bacteriological quali-ty before re-opening factories after workers’ strike.

## ACKNOWLEDGEMENTS

The authors thank the Joint Director (PH and CD), Department of Health, Director, NICED, Kolkata, Chief Health Officer, KMC, Chief Medical Officer of Health, South-24 Parganas, Executive Health Officer, Borough–IX KMC, Surveillance Medical Officer, IDSP, KMC, and the Water Analysist, KMC, for their support in this investigation. The authors acknowledge the help provided by Mr. Sailesh Singh in data collection.
